# “Back to the future”: toward Luria's holistic cultural science of human brain and mind in a historical study of mental retardation

**DOI:** 10.3389/fnhum.2013.00509

**Published:** 2013-09-03

**Authors:** Eli Lamdan, Anton Yasnitsky

**Affiliations:** ^1^Department of General History and the Program for History, Philosophy, and Sociology of Science, Technology, and Medicine, The Hebrew University of JerusalemJerusalem, Israel; ^2^Unaffiliated ResearcherToronto, ON, Canada

**Keywords:** Alexander Luria, cultural neuropsychology, developmental neuropsychology, defectology, international congress, International Association for Child and Adolescent Psychiatry and Allied Professions (IACAPAP)

The numerous successes of contemporary neuroscience and neuropsychology are obvious and undeniable. Brain research is clearly on the rise these days, and brain-related studies are rapidly growing in numbers and expanding in newer cross-disciplinary fields such as brain, mind, and… education, computation, consciousness, memory, cognition, behavior, culture, the arts, creativity, and the like. This special issue of *Frontiers in Human Neuroscience* is dedicated to yet another interesting topic: the interplay between brain research and special education, its theory and practice.

In search of a historical precedent and the cultural prototype of contemporary acute interest in the interplay between neuroscience and special education we discover an author well known among general readers and, like other pioneers of brain research, among the neuroscience academics: the Russian scholar Alexander Luria (1902–1977). The neurologist and bestselling author Oliver Sacks, a great admirer of both Luria's scholarly and case-based idiographic writings, discussed the secret of Alexander Luria's success as the unusual combination of complementary “classic” and “romantic” approaches in the latter's research, practice, and thinking (Cole et al., 2013; Sacks, in press). Yet, such a description remains somewhat incomplete unless one adds to it yet another utterly important dimension: Luria's social activism in his youth, the transformative stance of his research, and cultural and holistic underpinnings of his theory of human biosocial and cultural-historical psychoneurological development—the theory that he launched decades ago working hand-in-hand with another luminary and pioneer of human sciences, Lev Vygotsky (1896–1934), the prominent cultural and Marxist psychologist. These social and cultural dimensions of Luria's approach are a considerable addition to his holism of the kind of a combination of “classical and romantic science” that does not necessarily exceed the natural borders of an organism as seemingly isolated from historically evolving social, cultural, and psychological environment (Proctor, 2011). Such a “higher order,” “cultural holism” of Luria's approach still further empowers his theory in its effort to deal with a range of issues and problems of practical and applied nature, and thus appears to be of immense interest to contemporary scholars and practitioners.

In sum, Luria appears highly relevant to the topic of our present discussion of the neurological approach to special education in its search of identity. Little is known nowadays about Luria's period of transformation from cultural psychologist of Vygotskian type to the neuropsychologist as we know him now. Even less known is the fact that at certain point in his early career in 1930s Luria became a professional who could be best described as a neurologically-inclined *defectologist*, or, in somewhat more contemporary and politically correct parlance, a specialist in special education with background and research interest in neuropsychology. Thus, Luria can perhaps be referred to as one of the first neuropsychologists in the field of special education. In this paper we would like to focus on a snapshot of Luria's earlier career in order to discuss a concrete example of Luria's first neuro-defectological work of the kind that remains virtually unknown until today.

This work was summarized in the paper that Luria prepared for the presentation at the First International Congress of Child Psychiatry in Paris in 1937, the scientific event that became the inaugural meeting for the contemporary International Association for Child and Adolescent Psychiatry and Allied Professions (IACAPAP), the most recent Congress of which took place in 2012, yet again in Paris. Luria—most likely for political reasons—never made the trip and, therefore, the paper was presented in the author's absence. Luckily, the paper came out in French in the proceedings of the Congress and is available to us (Luria, 1937). To the best of our knowledge this is the first ever substantial discussion of this Luria paper in English.

It is truly remarkable how Luria's presentation appears to be up to date in a number of points. Thus, Luria opens his report expressing dissatisfaction with contemporary state of knowledge on the problem of mental retardation. In the absence of a neurological theory of this phenomenon, research focused on description and classification, based on broad statistical tests, such as the IQ test. Luria warns us that such an approach only distances us from truly understanding the phenomenon. The greatest danger it poses is confusion between retardation resulting from social circumstances and that resulting from organic defects. He claims that the solution is in-depth clinical case studies of the development of these defective states, using pathopsychological and pathophysiological methods.

Luria criticizes even those attempts that try to offer a neurological or psychological explanation to the problem of mental retardation. He argues that most of them are trying to identify in a direct way the relationship between a particular disease and a dysfunction of a specific neuropsychological system (like perception, emotions, etc.).

Luria, in contrast, understands the problem of mental retardation through the developmental prism. He sees it not as a disease of some system that performs a specific psychological function, but as a defect that took place during development. Because all mental functions are developmentally interrelated, it becomes necessary to understand normal development in order to reach a comprehensive theoretical understanding of mental retardation. It also follows that damage to any neuropsychological aspect during development and afterwards will conceivably present with very different results.

Luria provides concrete examples of the clinical studies that support his developmental approach to mental retardation. First, Luria deals with a case of a 14 years old patient, Fedia K., who fell ill with encephalitis at the age of 8 months, and whose mother realized after some time that he could not see. The examination showed that his basic disorder was visual agnosia, but its effect was very extensive. Fedia had a rich vocabulary and an excellent memory for words, but his speech was completely void—i.e., asemantic, meaningless—and consisted mainly of the reproduction of common speech patterns. The verbal formulas that he used did not make any sense, and were not related to objects in question. His behavior, intellectual activity and inner world were detached from reality and totally meaningless. All this is very different from cases of visual agnosia in adults, in which, despite widespread systemic damage, the patients were able to compensate some of their functions, for example, through the use of language, and did not reach the state of dementia. The explanation for this difference is the fact that perception, especially visual, is used as a basis for further development of the entire psychological system of the child. Damage to this psychological process at such an early stage disrupts the whole development and leads to an oligophrenic state.

The second case study that Luria deals with in his report is a case of five years old identical twins Yura and Lyosha Sh. The brothers had a speech impediment, probably hereditary, later diagnosed as a result of acoustic agnosia. They hardly spoke at all. They used several primitive sounds and their meaning changed depending on a particular situation. In general, their behavior was more suited to the developmental level of two years old, than their peers. They played mostly primitive games, could not draw or build a mosaic, lacked imagination and did not understand imaginary situations. Later, after an educational and corrective intervention that included separation of the twins and linguistic-acoustic training, the situation greatly improved. Luria argues that their mental retardation disappeared after ten months and they were left only with a lisp. Again, there is a marked difference between the situation of the twins and the situations of acoustic agnosia caused in an older age.

From such case studies Luria draws two main conclusions about the nature of mental retardation. First, they support his hypothesis that the effect from damage of the same function is quite different if that person is an adult or a child. And more importantly, they show that the effect depends not on the function itself, but on the role that it plays at that particular time for the future development of various functional psychological systems. Luria concludes that these findings corroborate Vygotsky's insight that the oligophrenic states such as idiocy, imbecility, debility, are diseases of centers whose localization in brain is not identical and, therefore, occur at different times.

At the end of his report Luria argues that these clinical, *defectological* findings lead him to treat a broader, *neurological* theoretical issue—the problem of cerebral localization. They support Vygotsky's hypothesis that the effect of a local lesion which took place during childhood is determined primarily by the role that this cerebral center plays in the future development of psychological processes. Accordingly, Luria presents a more complex approach on the cerebral localization of functions. He did neither see mental functions as tightly localized in particular brain areas, nor as the product of brain activity “as a whole.” Luria's approach considers various brain regions as components of functional systems that are involved in complex psychological functions. Therefore, damage in a particular brain area should be judged by its role in the functional system and the functional system's role in the future development of the individual. And this is how Luria ends his “neuro-defectological” paper, the first of its kind.

A deeper analysis of this paper as well as the entire system of Luria's cultural and holistic “neuro-defectology” is outside the scope of this small opinion essay. Perhaps not all aspects of this system are equally well discussed and, thus, remain only implicit in this specific presentation—like, for instance, Luria's political, emancipatory stance and his firm belief in virtually boundless potential of educational, rehabilitative, and correctional social practice. Another notable omission is the Vygotsky-Luria's theory of meaning and consciousness that is not integrated into this conceptual framework, which seems to present one of the most important future tasks and constitutes the “zone of proximal development” for this theory in its present form.

Nevertheless, even a sketchy account of Luria's cultural, neurological, and developmental perspective on the problem of disability and abnormal development gives us a sense of the breadth of Lurian science, capable of dealing with a really impressive range of topics. This is, perhaps, the most important lesson we still need to learn from the work of the Soviet scholars such as Vygotsky, Luria, and their associates. And still, here lies the major problem with the scientific legacy of these classic world-known authors: not only to see their theory in all its integrative power of a Soviet idiosyncratic “historical-materialist” holistic and dialectical theory of the “Mozart” and the “Beethoven of psychology” (Toulmin, 1978), but also not to miss the relatively less known and underdeveloped, yet possibly equally promising opportunities that it offers to contemporary scholars. One needs to understand that this is not a “fossilized” intellectual construct, but a dynamic system of thought, “pregnant” with exciting possibilities of further theoretical, practical and applied development such as, for instance, the unfinished Vygotsky-Luria's theory of meaning, sense-making, and consciousness.

Perhaps, it is from this scholarship that our contemporary science will borrow in its search of an integrative conception that would equally well operate in terms of strictly “classical” research and “romantic” science as seen from a holistic, historical, and integrative socio-cultural and developmental perspective, still not fully understood to date.
